# RETRACTION: Efficacy and Safety of Dexmedetomidine as an Adjuvant to Local Wound Infiltration Anaesthesia: A Meta‐Analysis With Trial Sequential Analysis of 23 Randomised Controlled Trials

**DOI:** 10.1111/iwj.70645

**Published:** 2025-04-23

## Abstract

**RETRACTION:** RenY. , WeiM. , LiuH. , WangY. , ChenH. , LiZ. , ShiW. , and YouF. , “Efficacy and Safety of Dexmedetomidine as an Adjuvant to Local Wound Infiltration Anaesthesia: A Meta‐Analysis with Trial Sequential Analysis of 23 Randomised Controlled Trials,” International Wound Journal 18, no. 1 (2021): 32–48, 10.1111/iwj.13517.33169515 PMC7949019

The above article, published online on 9 November 2020, in Wiley Online Library (http://onlinelibrary.wiley.com/), has been retracted by agreement between the journal Editor in Chief, Professor Keith Harding; and John Wiley & Sons Ltd. Following an investigation by the publisher, all parties have concluded that this article was accepted solely on the basis of a compromised peer review process. The editors have therefore decided to retract the article.

**RETRACTION:** Y. Ren , M. Wei , H. Liu , Y. Wang , H. Chen , Z. Li , W. Shi , and F. You , “Efficacy and Safety of Dexmedetomidine as an Adjuvant to Local Wound Infiltration Anaesthesia: A Meta‐Analysis with Trial Sequential Analysis of 23 Randomised Controlled Trials,” International Wound Journal 18, no. 1 (2021): 32–48, 10.1111/iwj.13517.33169515 PMC7949019

The above article, published online on 9 November 2020, in Wiley Online Library (http://onlinelibrary.wiley.com/), has been retracted by agreement between the journal Editor in Chief, Professor Keith Harding; and John Wiley & Sons Ltd. Following an investigation by the publisher, all parties have concluded that this article was accepted solely on the basis of a compromised peer review process. The editors have therefore decided to retract the article.

